# Association Between BMI Reduction and Activities of Daily Living Improvement in Elderly Patients With Musculoskeletal Disorders in a Convalescent Rehabilitation Ward

**DOI:** 10.7759/cureus.71724

**Published:** 2024-10-17

**Authors:** Isao Uno

**Affiliations:** 1 Rehabilitation Medicine, Sakura Juji Fukuoka Hospital, Fukuoka, JPN

**Keywords:** adl (activities of daily living), body mass index: bmi, elderly individuals, musculoskeletal conditions, rehabilitation medicina

## Abstract

Background

As populations age, musculoskeletal disorders significantly affect the quality of life (QOL) and functional independence of elderly individuals. While BMI is commonly used to assess nutritional status and is linked to physical function, the relationship between BMI reduction and improvements in activities of daily living (ADL) remains unclear in elderly patients with musculoskeletal disorders. This study aimed to explore this relationship in a rehabilitation setting.

Methods

This retrospective cohort study included 75 elderly patients (65 years and older) with musculoskeletal disorders admitted to a convalescent rehabilitation ward. Patients were categorized into two groups based on BMI reduction: those with a decrease of ≥0.7 kg/m² and those with <0.7 kg/m². Functional Independence Measure (FIM) scores were recorded at admission and discharge. Statistical analyses, including logistic regression, were conducted to examine the relationship between BMI reduction and FIM improvement.

Results

There was no significant difference in FIM gain between the BMI-reduced and non-BMI-reduced groups. Logistic regression analysis also found no significant association between BMI reduction and FIM improvement, even after adjusting for age, gender, length of stay, and initial FIM scores.

Discussion

The absence of a significant association may be due to factors such as postoperative swelling, comorbidities, and the predominance of female participants, who may experience less muscle mass loss. Study limitations include the lack of data on dietary intake and body composition. Future research should include broader assessments, such as muscle mass and swelling, to better evaluate rehabilitation outcomes in elderly patients with musculoskeletal disorders.

## Introduction

With the aging population in many countries, including Japan, health issues specific to the elderly have become increasingly important. Among these, musculoskeletal disorders are major concerns that significantly affect the quality of life (QOL) and functional independence of older adults. According to a national survey in Japan, the estimated prevalence of musculoskeletal disorders in the elderly is 50-80% for knee osteoarthritis and 75-90% for lumbar osteoarthritis [1], making these conditions highly prevalent among degenerative diseases. The annual incidence of fractures per 100,000 people is reported as follows: 217 men and 567 women for proximal femur fractures, 82 men and 432 women for distal radius fractures, 26 men and 96 women for proximal humerus fractures, and 412 men and 1,229 women for vertebral fractures [2]. These statistics indicate that a substantial proportion of elderly individuals suffer from musculoskeletal disorders, leading to functional decline.

Functional decline due to bone fractures and musculoskeletal diseases in the elderly significantly reduces their ability to perform activities of daily living (ADL), increases the need for nursing care and medical assistance, and ultimately shortens healthy life expectancy [3]. Furthermore, the prevalence of malnutrition or risk of malnutrition is notably high among elderly patients with musculoskeletal conditions. For instance, the proportion of patients with malnutrition or at risk of malnutrition upon admission is reported to be 86% for those with proximal femur fractures [4], 61.1% for those with vertebral fractures [5], and 8.5-50% for those undergoing knee replacement surgery [6]. This suggests that older patients with musculoskeletal conditions are more likely to be undernourished, which can exacerbate their health outcomes. In elderly populations, poor nutritional status is associated with adverse outcomes, including reduced life expectancy, increased pain [7], higher rates of rehospitalization [8], and declines in ADL [9] and physical function [10]. In other words, the elderly, particularly those with musculoskeletal diseases, frequently experience malnutrition, which negatively impacts their health and functional capacity.

BMI, calculated by dividing weight by the square of height, is a widely used measure to assess nutritional status [11]. BMI is associated with body fat mass and skeletal muscle mass and serves as a useful indicator of risk for chronic diseases [12]. A high BMI is often correlated with a higher lean mass index, a measure of muscle mass, and better physical function [13]. Therefore, a decrease in BMI is typically accompanied by a reduction in lean mass, which may lead to a decline in physical function.

Given this context, managing BMI is crucial in post-stroke rehabilitation, as a decrease in BMI may hinder the recovery of physical function and ADLs. However, the relationship between BMI and ADL in patients with musculoskeletal diseases remains unclear, and there is limited evidence on the impact of BMI reduction on ADL in these patients. The purpose of this study was to clarify the relationship between BMI reduction and ADL improvement in elderly patients with musculoskeletal diseases admitted to a convalescent rehabilitation ward.

## Materials and methods

Participants

This is a retrospective cohort study in which data were collected from medical records. The subjects were patients aged 65 years or older with musculoskeletal disorders who were admitted to the convalescent rehabilitation ward between April 1, 2023, and March 31, 2024. Exclusion criteria included patients with missing endpoint data, those discharged due to death, or those urgently transferred due to a worsening condition. Patients began individual rehabilitation with physical and occupational therapists on the day of admission, with sessions lasting two hours per day for patients aged 89 years and younger, and 80 minutes per day for patients aged 90 years and older. All patients participated in a multidisciplinary conference within one week of admission to discuss goal setting and treatment planning.

Measurements

Information on age, gender, disease name, length of stay, discharge destination, BMI, and Functional Independence Measure (FIM) scores were obtained from medical records. BMI was calculated from height and weight at admission and discharge using the formula: weight (kg) / height (m)². The FIM is a measure of independence in daily living, scored on a seven-point scale ranging from 1 point ("total assistance") to 7 points ("complete independence"). It consists of 13 items related to motor function and 5 items related to cognition, with a maximum score of 126 and a minimum score of 18 [14]. FIM scores were assigned by the therapist responsible for each patient at both admission and discharge; the FIM was scored separately for motor FIM, cognitive FIM, and total FIM. FIM improvement was calculated as the FIM gain, by subtracting the admission FIM from the discharge FIM.

The cut-off value for BMI reduction was 0.7 kg/m², the minimal clinically important difference (MCID) for BMI change used in a national survey of convalescent rehabilitation wards [15]. Based on this cutoff value, patients with a decrease in BMI of 0.7 kg/m² or more were categorized into the BMI-reduced group, while those with a decrease of less than 0.7 kg/m² were categorized into the non-BMI-reduced group.

Statistical analysis

The normality of all survey items between the BMI-reduced and non-BMI-reduced groups was assessed using the Shapiro-Wilk test. For normally distributed data, an unpaired t-test was conducted; for non-normally distributed data, the Mann-Whitney U test was used. Additionally, a chi-square test was performed for binary variables. To clarify the effect of BMI reduction on FIM improvement, logistic regression analysis was conducted with the presence or absence of an FIM gain equal to or greater than the MCID [16] as the dependent variable. The explanatory variables included the presence or absence of BMI reduction, age, sex, length of stay in the ward, and FIM at admission. Statistical analysis was performed using EZR version 1.52 (Saitama Medical Center, Jichi Medical University, Saitama, Japan), with a significance level set at p < 0.05.

Ethical consideration

This study was approved by the Clinical Research Ethics Committee of Sakura Juji Fukuoka Hospital (approval number: SJF-2024-070801).

## Results

During the study period, there were 231 patients with musculoskeletal diseases. After excluding those who met the exclusion criteria, 75 subjects (61 women, median age 86 (79.5-90.0) years) were included in the study. Of these, 12 subjects were in the BMI reduction group (11 women, median age 87.5 [84.0-90.25] years), and 63 subjects were in the non-BMI reduction group (50 women, median age 86.0 (78.5-90.0) years). In the comparison between the groups, no significant differences were found between the BMI reduction group and the non-BMI reduction group for any of the items (p > 0.05) (Table 1).

**Table 1:** Patient characteristics. FIM: Functional Independece Measure; MCID: Minimal Clinically Important Difference. Data are presented as means ± SDs, as median (25th-75th percentiles), or as numbers (%). a: Student's t test b: Chi-squared test c: Mann-Whitney U test.

Item	Total (n=75)	BMI Reduction Group (n=12)	Non-BMI Reduction Group (n=63)	P-value
Age (years)	86 (79.5-90)	87.5 (84.0-90.25)	86.0 (78.5-90.00)	0.195^a^
Gender (n)				0.444^b^
Male	14 (18.7%)	1 (8%)	13 (21%)	
Female	61 (81.3%)	11 (92%)	50 (79%)	
Length of stay (days)	76 (64.5-86)	79 (78.5-88)	72 (64.0-86)	0.479^a^
Discharge destination				0.069^c^
Home	39 (52%)	5 (42%)	34 (54%)	
Residential facility	19 (25%)	3 (25%)	16 (25%)	
Nursing home	2 (3%)	2 (17%)	0 (0%)	
General ward	5 (7%)	1 (8%)	4 (6%)	
Long-term care ward	1 (1%)	1 (8%)	0 (0%)	
Other rehabilitation hospital	2 (3%)	0 (0%)	2 (3%)	
Other	7 (10%)	0 (0%)	7 (11%)	
Disease classification				0.668^c^
Femoral fracture	44 (59%)	6 (50%)	38 (60%)	
Spinal fracture	19 (25%)	5 (42%)	14 (22%)	
After joint replacement:	10 (13%)	1 (8%)	9 (15%)	
Other	2 (3%)	0 (0%)	2(3%)	
BMI at admission	21.1 (18.5-22.6)	21.25 (20.50-23.425)	20.70 (18.35-22.450)	0.618^b^
BMI at discharge	20.8 (18.2-23.4)	20.45 (17.7-22.3)	20.90 (18.4-23.4)	0.406^b^
FIM at admission	68 (49.5-84)	65.5 (58.0-74.75)	68.0 (47.5-84.50)	1^a^
Motor FIM at admission	42 (29-51.5)	40 (29.25-45)	42 (29.00-53)	0.479^a^
Cognitive FIM at admission	26 (19-31.5)	29 (24.5-32.5)	25 (18.0-32.5)	0.249^a^
FIM at discharge	101 (79.5-116.5)	97 (85.75-108.25)	101 (78.00-117.00)	0.806^a^
Motor FIM at discharge	72 (52.5-83.5)	68.5 (55.5-76.25)	73.0 (52.0-84.00)	0.506^a^
Cognitive FIM at discharge	30 (24-34)	31 (26.0-35)	30 (20.5-34)	0.493^a^
FIM Gain	28 (18.5-39)	20 (14.25-47.75)	29 (19.00-38.00)	0.654^a^
Motor FIM gain	25 (15.5-35)	18.5 (15.0-44.0)	27.0 (16.5-34.5)	0.834^a^
Cognitive FIM gain	1 (0-4)	2 (0-3.25)	1 (0-4.5)	0.621^a^
FIM gain MCID (n)				0.116a
Improvement	47 (63%)	5 (42%)	42 (67%)	
No improvement	28 (37%)	7 (58%)	21 (33%)	
Motor FIM gain MCID (n)				0.163a
Improvement	53 (71%)	6 (50%)	47 (74%)	
No improvement	22 (29%)	6 (50%)	16 (26%)	

In the logistic regression analysis, only age was significantly associated with an improvement in FIM gain exceeding the MCID (p < 0.05), while no significant associations were observed for other factors in relation to FIM gain exceeding the MCID (p > 0.05) (Table 2).

**Table 2:** Factors associated with FIM gain: a logistic regression analysis. FIM: Functional Independece Measure; CI: Confidence interval. *=p<0.05

	Odds ratio	95% CI Lower	95% CI Upper	P-value
Age	0.915	0.847	0.988	0.024*
Gender	1.28	0.281	5.87	0.747
FIM at admission	0.995	0.969	1.02	0.711
BMI reduction	2.24	0.597	8.42	0.231
Length of stay	0.994	0.966	1.02	0.706

## Discussion

This study examined the effect of BMI reduction on FIM gain in elderly patients with musculoskeletal conditions in a recovery unit and found no significant association. Three factors may explain this result.

The first factor that may have contributed to the lack of effect of BMI reduction on FIM improvement is the presence of swelling due to fractures or surgery, which can affect BMI. In postoperative patients with proximal femur fractures, swelling increases the volume of the operated limb for up to 30 days, peaking at seven days postoperatively [17]. In patients with ankle fractures, residual swelling has been reported to persist for 3-6 months after injury [18]. As swelling decreases over time and rehabilitation progresses, this reduction in swelling can lead to a decrease in BMI. Swelling is associated with decreased muscle strength, postural control, and basic movement ability, while physical function improves as swelling subsides [19]. In other words, ADLs may improve if BMI reduction is due to decreased swelling rather than worsening nutritional status. Therefore, the lack of an association between BMI reduction and FIM improvement in patients with musculoskeletal diseases may be because the study population included both patients whose BMI decreased due to worsening nutritional status and those whose BMI decreased due to reduced swelling.

Second, it is possible that factors not investigated in this study significantly impacted FIM gain. For example, in patients with fractures, physical function often improves over time as the injured site heals, regardless of whether the fracture was treated surgically or conservatively [20]. Although we were unable to investigate the length of stay at the acute care hospital in this study, it is possible that the number of days since the onset of the disease or surgery allowed for progress in tissue repair and improvement in ADLs. Another potential factor is the influence of comorbidities. Approximately 30% of community-dwelling elderly people have multimorbidity, and those with multimorbidity tend to have lower energy and protein intake [21]. Multimorbidity is also associated with sarcopenia [22] and falls [23]. Although we were unable to investigate comorbidities in this study, it is likely that the subjects were affected by multimorbidity due to their advanced age and the high incidence of fractures from falls. Additionally, the mental and psychological state of the subjects may have influenced the results. Among the elderly, about 35% have depressive symptoms, and those with depressive symptoms tend to have lower ADLs [24]. The presence of depressive symptoms also increases the risk of lower ADL independence later in life [25]. Given the age of the participants, it is plausible that a significant number of them experienced depressive symptoms, which may have affected FIM gain.

Third, there may have been a sampling bias in this study. A larger proportion of the subjects were female, with fewer males. A study of elderly inpatients reported that muscle mass and body fat mass were associated with ADL and IADL only in men, with no significant association found in women [26]. In elderly populations, a greater loss of muscle mass with weight loss has been reported in men compared to women [27,28]. Muscle mass correlates with muscle strength [29] and physical function [30]. In this study, women accounted for approximately 80% of the subjects. This large proportion of women may have resulted in a higher number of subjects with a smaller decrease in muscle mass relative to BMI reduction, which could explain the lack of association between BMI reduction and ADL improvement. Additionally, the limited sample size in this study, particularly the small number of subjects in the BMI reduction group, may have influenced the results. The BMI reduction group comprised only 16% of the total sample. Although no significant differences were observed between the groups in the survey data, and the sample characteristics were similar, the large disparity in sample sizes could have contributed to the lack of significant findings.

Finally, it is essential to consider the patient's oral function. In older adults, a low BMI is often associated with impaired oral function, which can restrict the range of foods that can be consumed [31]. Additionally, older adults with a reduced BMI are at heightened risk for compromised swallowing function, or dysphagia [32]. Poor oral health diminishes the ability to chew and adequately process food, thereby exacerbating nutritional deficiencies and contributing to muscle loss [33]. Similarly, impaired swallowing function limits food intake, further increasing the risk of malnutrition [34]. Consequently, both oral and swallowing dysfunctions may contribute to a reduction in BMI and negatively influence the acquisition of functional independence as measured by FIM. Future research should account for the potential impact of these factors on BMI reduction and functional outcomes.

Limitations

There are three limitations to this study. First, it did not investigate dietary intake or nutritional support status. Changes in dietary intake affect BMI, and the presence or absence of nutritional support interventions also influences BMI changes. Second, we were unable to examine body composition, which is influenced by changes in muscle mass, fat mass, and water content, all of which contribute to BMI loss. Third, as this was a retrospective cohort study, there may have been limitations in the completeness and consistency of the data collection, which relied on existing medical records. Some subjects were excluded due to missing data, and several variables could not be investigated. Therefore, when interpreting the results, it is important to consider that some participants were excluded.

## Conclusions

The present study suggests that BMI reduction was not associated with ADL in elderly patients with musculoskeletal diseases. This finding indicates that changes in weight and BMI alone are insufficient for nutritional assessment in rehabilitation, highlighting the need for a multidimensional evaluation that includes changes in postoperative swelling, muscle mass, and other nutritional indices. Future research should include prospective studies with larger sample sizes and incorporate physical indicators other than BMI, such as swelling and muscle mass, to more accurately assess the effectiveness of rehabilitation in patients with musculoskeletal disorders.
